# Body Composition Assessment by Air-Displacement Plethysmography Compared to Dual-Energy X-ray Absorptiometry in Full-Term and Preterm Aged Three to Five Years

**DOI:** 10.3390/jcm11061604

**Published:** 2022-03-14

**Authors:** Inge A. L. P. van Beijsterveldt, Victoria A. A. Beunders, Alja Bijlsma, Marijn J. Vermeulen, Koen F. M. Joosten, Anita C. S. Hokken-Koelega

**Affiliations:** 1 Department of Pediatrics, Subdivision of Endocrinology, Erasmus Medical Center-Sophia Children’s Hospital, 3015 CN Rotterdam, The Netherlands; a.hokken@erasmusmc.nl; 2 Department of Pediatrics, Division of Neonatology, Erasmus Medical Center-Sophia Children’s Hospital, 3015 CN Rotterdam, The Netherlands; v.beunders@erasmusmc.nl (V.A.A.B.); a.bijlsma@erasmusmc.nl (A.B.); m.j.vermeulen@erasmusmc.nl (M.J.V.); 3 Department of Pediatric Intensive Care, Sophia Erasmus Medical Center-Sophia Children’s Hospital, 3015 CN Rotterdam, The Netherlands; k.joosten@erasmusmc.nl

**Keywords:** ADP, DXA, fat-free mass density, Dffm estimates

## Abstract

It is important to monitor body composition longitudinally, especially in children with atypical body composition trajectories. Dual-energy X-ray absorptiometry (DXA) can be used and reference values are available. Air-displacement plethysmography (ADP) is a relatively new technique, but reference values are lacking. In addition, estimates of fat-free mass density (Dffm), needed in ADP calculations, are based on children aged >8 years and may not be valid for younger children. We, therefore, aimed to investigate whether DXA and ADP results were comparable in young children aged 3–5 years, either born full-term or preterm, and if Dffm estimates in the ADP algorithm could be improved. In 154 healthy children born full-term and 67 born < 30 weeks of the inverse pressure-volume gestation, aged 3–5 years, body composition was measured using ADP (BODPOD, with default Lohman Dffm estimates) and DXA (Lunar Prodigy). We compared fat mass (FM), fat mass percentage (FM%) and fat-free mass (FFM), between ADP and DXA using Bland–Altman analyses, in both groups. Using a 3-compartment model as reference method, we revised the Dffm estimates for ADP. In full-term-born children, Bland–Altman analyses showed considerable fixed and proportional bias for FM, FM%, and FFM. After revising the Dffm estimates, agreement between ADP and DXA improved, with mean differences (LoA) for FM, FM%, and FFM of −0.67 kg (−2.38; 1.04), −3.54% (−13.44; 6.36), and 0.5 kg (−1.30; 2.30), respectively, but a small fixed and proportional bias remained. The differences between ADP and DXA were larger in preterm-born children, even after revising Dffm estimates. So, despite revised and improved sex and age-specific Dffm estimates, results of ADP and DXA remained not comparable and should not be used interchangeably in the longitudinal assessment of body composition in children aged 3–5 years, and especially not in very preterm-born children of that age.

## 1. Introduction

Childhood obesity tracks into adulthood and has been linked to both short-term and long-term morbidity [1,2]. Consequently, it is important to identify children at risk of excess adiposity early in childhood in order to start preventive and therapeutic strategies as early as possible. Body composition is a more adequate indicator of adiposity than standard anthropometric measures such as weight or body mass index (BMI), especially in infants and young children [3,4]. Therefore, reliable methods to longitudinally assess body composition from early childhood onwards are needed. Specific attention should be given to children at risk of altered adiposity trajectories, such as children born preterm [5].

Multiple tools are available for measuring body composition during childhood, with Dual-energy X-ray Absorptiometry (DXA) and Air-Displacement Plethysmography (ADP) being most frequently used [6,7]. DXA is often used as a reference method to determine body composition in research and clinical practice [7]. Longitudinal reference values are available for infants and young children from birth until age 5 years, showing slightly higher fat mass in girls as compared to boys [8,9]. However, DXA uses a very small dose of radiation (0.0002 mSv). ADP calculates body composition by measuring body volume, using the inverse pressure-volume relation [10] and can be applied in infants ≤ 6 months old and/or ≤8 kg using PEAPOD [11,12], and in children ≥ 2 years and ≥12 kg using BODPOD [13]. ADP is, however, currently more costly than DXA and requires the cooperation of the child, as movement and crying influence results [13]. It is our experience that BODPOD is feasible in children ≥ 3 years of age. Importantly, ADP uses multiple assumptions to calculate body composition parameters from measured body volume. These assumptions include estimates for fat-free mass (FFM) density (Dffm). The default estimates in BODPOD software are based on outdated, small studies in which results of healthy older children and adults were extrapolated to children aged <8 years, per 2-year intervals [14,15]. These Dffm estimates may, therefore, not be valid in young children. In fact, especially in young children with deviant body composition, such as preterm-born children, we noticed that ADP results are often clinically questionable (e.g., extremely low values of fat mass percentage (FM%), <5%). Wells et al. developed novel Dffm estimates for healthy children aged ≥5 years [16], which have been reported to be superior to the default estimates in ADP for children aged 5 years [17]. However, improved Dffm estimates for children aged <5 years are not yet available.

In order to monitor body composition longitudinally in infants and young children, it would be favorable if ADP and DXA could be used interchangeably. Our research group reported that results from ADP (PEAPOD) were comparable with DXA in infants aged 6 months [8]. Studies in healthy school children, adolescents, and adults, however, showed conflicting results on comparability [10,18,19,20]. In young children, aged 3–5 years, comparison between ADP and DXA has not yet been described.

The primary aim of our study was to compare fat mass (FM), FM%, and FFM results assessed by ADP with DXA, in a cohort of healthy full-term-born children aged 3 to 5 years. Secondly, we aimed to explore potential improvements to the default Dffm estimates in the ADP algorithm for full-term-born children in this age category. Furthermore, we evaluated body composition based on the default and revised Dffm estimates in a group of very preterm-born children aged 3–5 years. We hypothesized that ADP and DXA are both reliable methods to estimate body composition in young children, but may not be used interchangeably, especially not in very preterm-born children aged 3–5 years.

## 2. Material and Methods

### 2.1. Study Setting and Subjects

The current cross-sectional study included subjects of two ongoing prospective birth cohort studies on growth and body composition, which started from 2012 at the Erasmus MC Sophia Children’s Hospital in Rotterdam, The Netherlands. The Sophia Pluto study included healthy full-term born infants [21,22], whereas the BOND study included infants born very preterm (<30 weeks gestation) [23]. The full-term born infants (≥37 weeks) were recruited from all seven maternity wards in Rotterdam and experienced an uncomplicated neonatal period. Infants with a complicated perinatal or neonatal period were excluded: in case of maternal disease or medication that could interfere with growth and development, perinatal asphyxia, neonatal sepsis, neonatal respiratory ventilation, and significant congenital or intrauterine disease [21]. The very preterm-born children were admitted to our level IV neonatal intensive care unit within 48 h after birth. Exclusion criteria for this group included congenital and chromosomal anomalies that could interfere with growth, severe brain injury, congenital infection, or perinatal asphyxia [23].

The present analyses were based on a subgroup of children from both cohorts whose body composition was, per study protocol, measured by both ADP and DXA at the age of 3–5 years, between April 2019 and November 2021. All participants of both cohorts who were in this age range within this timeframe were eligible. The Medical Ethics Committee of the Erasmus MC approved both studies (MEC-2012-164 and MEC-2014-379). We obtained written informed consent of all parents/caregivers.

### 2.2. Data Collection and Measurements

For full-term-born subjects, outpatient clinic visits were scheduled at 3, 4, and 5 years, and for preterm-born subjects at 3 and 5.5 years corrected age. Data on child ethnicity were derived from parental questionnaires.

### 2.3. Anthropometrics

Weight was measured without heavy clothing to the nearest 5 g using a flat scale (Seca, Hamburg, Germany). Height was measured twice to the nearest 0.1 cm in upright position by a stadiometer (Seca), with the average of both measurements used in the analyses. Age and sex-corrected SD-scores for weight and length at birth and at 3–5 years were calculated using Dutch reference values, and Fenton charts at preterm birth [24,25].

### 2.4. Body Composition

In 154 healthy full-term-born children and 67 very preterm children, body composition was measured by ADP and DXA within one hour. For ADP, we used BODPOD (COSMED) with pediatric hardware and software, including the default Lohman density model [14]. Children wore tight underwear (without diaper) and a Lycra cap covering all scalp hair [14]. The DXA (Lunar Prodigy, GE Healthcare) was used with Encore v14.1 software. During DXA scan, children wore light clothing. FFM was calculated as the sum of lean body mass and bone mineral content.

The same ADP and DXA devices were used during the entire study period. Both devices were calibrated daily, and used and maintained according to the supplier’s manuals [13]. During measurements, children were instructed not to move. We excluded measurements if the supplier’s terms of use were not met, or when the child cried. To test reliability, a random sample of full-term-born children was measured twice, after repositioning, with the same device (13 with ADP and 16 with DXA). Intra-class-correlation coefficients for FM, FM%, and FFM for ADP were 0.980, 0.978, and 0.994, and for DXA 0.991, 0.985, and 0.994 (all *p* < 0.001), respectively.

The BODPOD calculates FM% using two constants (C1 and C2), derived from the programmed, sex-specific density models for Dffm and FM density (Dfm), and measured body density (BD (kg/L)), as expressed in Formula (1) [14]:

C1 = (Dffm ∗ Dfm)/(Dffm − Dfm) C2 = Dfm/(Dffm − Dfm) FM% = (C1/BD − C2) ∗ 100% (1)

The standard algorithm in the BODPOD software follows the assumption that Dfm remains stable during life at 0.9007 kg/L [14,15,16]. Consequently, Formula (1) can be rewritten as Formula (2):

Dffm = ((0.9007 ∗ FM% − 90.07) ∗ BD)/(FM% ∗ BD − 90.07) (2)

### 2.5. Statistical Analysis

Children born full-term and preterm were analyzed as separate groups. Independent sample *t*-tests were used to compare group characteristics. Paired sample *t*-tests were used to compare ADP and DXA results for each group at all ages. Bland–Altman analyses were used to test agreement between ADP and DXA results. Fixed bias was determined by one sample *t*-test, and proportional bias by linear regression. As body composition, such as anthropometrics, differs per sex, we analyzed boys and girls separately [8,9].

As the current algorithms used in ADP are based on Dffm’s of children aged >8 years, which are extrapolated for younger ages [14], we re-calculated Dffm for each included full-term born child. We used Formula (2) with body density (BD) as measured by ADP, and FM% as derived from the 3-compartment model [26]. For the 3 compartments, we entered body volume (BV) measured by ADP (BODPOD), bone mineral content (BMC) measured by DXA and body weight (BW) measured by scale, as follows:

$$\mathrm{FM}\%=(\frac{6.386*\mathrm{BV}+3.961*\mathrm{BMC}-6.09*\mathrm{BW}}{\mathrm{BW}})*100\%$$

We used the re-calculated Dffm values to create sex-specific curves by age, using generalized additive models for location, scale, and shape (GAMLSS) [27,28]. Box-Cox Cole and Green distribution (BCCG) was applied to fit the three parameters of mu (µ), sigma (σ) and nu (ν). The distribution expresses the mean (µ), variance (σ), and skewness (ν) that change as a function of age. Median Dffm was then assessed for ages between 3 and 5 years, using 0.25-year time intervals. Based on these new sex- and age-specific median Dffm estimates, we re-calculated FM% for each ADP measurement using Formula (1). For children with age > 5 years, which was above the modelled age range, we used Wells et al. Dffm estimates [17].

A 2-tailed *p*-value < 0.05 was considered statistically significant. Analyses were performed using SPSS-package 25.0 (IBM SPSS Statistics, Armonk, NY, USA) and R with GAMLSS-package v.5.2.0 (V 4.0.0 for MacOS, R Core Team, Vienna, Austria).

## 3. Results

Clinical characteristics of the full-term and preterm-born children are shown in Table 1**.** SD-scores for weight-for-height and height were lower in the preterm compared to the full-term group at 3 and 5 years corrected age. Body composition parameters assessed by ADP and DXA are presented in Table 2. FM and FM%, assessed by DXA, were higher in full-term compared to preterm-born children at each time point (all *p* ≤ 0.001). In both groups, FM and FFM increased with age and body-size corrected FM% decreased with age (Supplemental Table S1).

### 3.1. Comparison between ADP and DXA in Full-Term-Born Children

**Figure 1:**
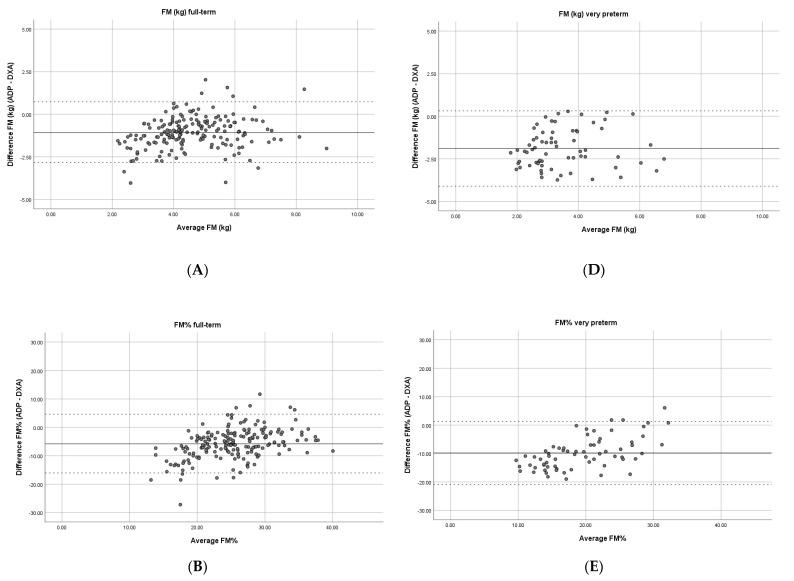

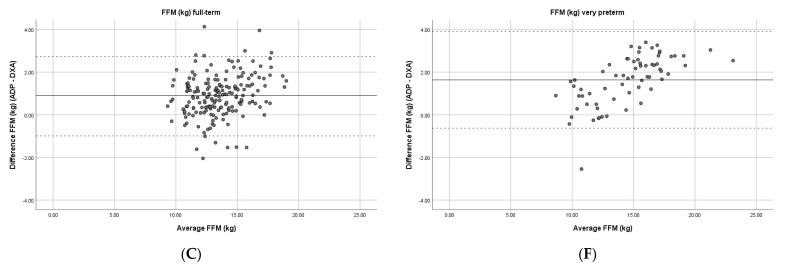
Bland–Altman plots for FM, FM%, and FFM measured by ADP and DXA in full-term (**A**–**C**) and very preterm-born children (**D**–**F**) aged 3–5 years: Continuous line represents the mean difference between ADP and DXA. The dashed lines represent the limits of agreement. Abbreviations: FM, fat mass, FM%, fat mass percentage, FFM, fat-free mass; kg, kilograms; DXA, dual-energy X-ray absorptiometry; ADP, air-displacement plethysmography.

Absolute results of FM, FM%, and FFM by ADP and DXA were significantly different (all, *p* < 0.001) (Table 2). Bland–Altman analyses (Figure 1) showed that mean differences (limits of agreement (LoA)) for FM, FM%, and FFM between ADP and DXA were −1.08 (−2.92; 0.76), −5.78% (−16.25; 4.69) and 0.90 kg (−1.00; 2.80), respectively. For all three parameters, a fixed bias (all, *p* < 0.001) and a proportional bias for FM (β: 0.135, *p* = 0.014), FM% (β: 0.396, *p* < 0.001), and FFM (β: 0.109, *p* = 0.002) were observed. Proportional bias indicates that the difference between ADP and DXA increased when the result deviated more from the mean.

### 3.2. Revised FFM Density Model

**Figure 2:**
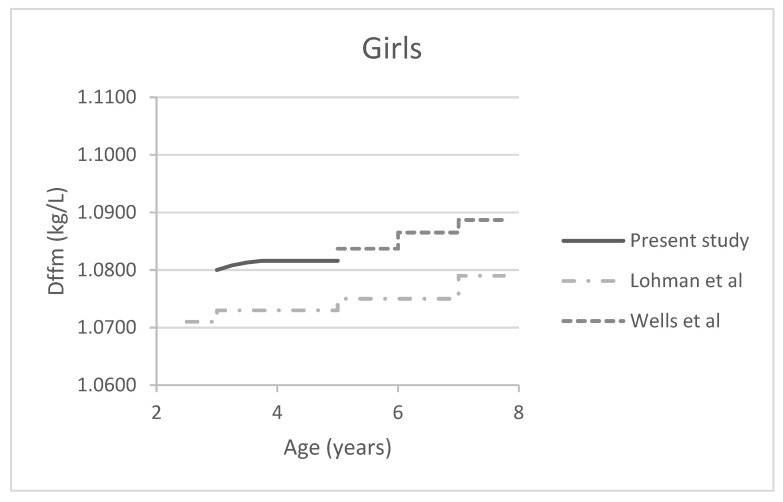

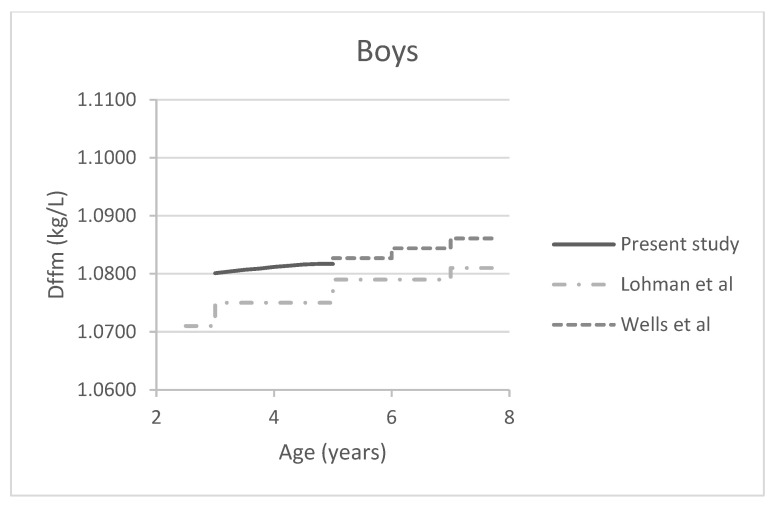
Dffm estimates plotted against age for boys and girls separately: Presented are the revised Dffm estimates from present study and those of Lohman et al. and Wells et al. [16]. Abbreviation: Dffm=fat-free mass density.

Table 3 presents the revised, sex-specific estimates for Dffm for full-term children aged 3–5 years. Dffm increased between age 3 and 5 years. Compared to the default Lohman Dffm model, the revised Dffm estimates are higher at all ages. At age 5 years, they were in the range of the Wells et al. [16] estimates (Figure 2).

The agreement with DXA improved when using the revised Dffm estimates for ADP, with mean differences (LoA) for FM, FM%, and FFM of −0.67kg (−2.38; 1.04), −3.54% (−13.44; 6.36) and 0.50 kg (−1.30; 2.30), respectively (Table 2). Although smaller, a fixed (all, *p* < 0.001) and proportional bias remained, for FM (β: 0.135, *p* = 0.010), FM% (β: 0.374, *p* < 0.001), and FFM (β: 0.106, *p* = 0.002).

### 3.3. Comparison between ADP and DXA in Very Preterm-Born Children

Using the default Dffm estimates in children born very preterm, absolute results of ADP and DXA were very different (all *p* < 0.001) ([Table jcm-11-01604-t002]). In fact, differences in FM, FM%, and FFM results between both methods were significantly larger in preterm compared to full-term-born children (all, *p* < 0.001), with mean differences (LoA) of −1.89 kg (−4.10; 0.32) for FM, −9.79% (−20.92; 1.34) for FM%, and 1.64 kg (−0.63; 3.91) for FFM (Figure 1). Similar to the full-term group, a fixed bias (*p* < 0.001) was observed for all three parameters, and a proportional bias for FM% (β: 0.575, *p* < 0.001) and FFM (β: 0.264, *p* = 0.001), but not for FM (β: 0.080, *p* = 0.504).

When using the revised Dffm estimates, comparison of ADP and DXA showed smaller fixed bias, with mean differences (LoA) for FM: −1.45 kg (−3.53; 0.63), FM%: −7.32% (−18.26; 3.62), FFM: 1.20 kg (−0.92; 3.32) (Table 2), but the proportional bias remained similar.

## 4. Discussion

To our knowledge, this is the first study to compare ADP with DXA in a relatively large group of young children aged 3–5 years who underwent both ADP and DXA. We observed significant differences in FM, FM%, and FFM results derived with both techniques. Based on our cohort of healthy full-term-born children, we provide a revised Dffm model to be used with ADP in children aged 3–5 years. Furthermore, differences between ADP, using default or revised Dffm estimates, and DXA were significantly larger in very preterm compared to full-term-born children. Although our revised Dffm estimates improved agreement between ADP and DXA, we have to conclude that results of both techniques are not comparable and should, thus, not be used interchangeably in the longitudinal assessment of body composition in children aged 3–5 years.

Literature on comparison of ADP and DXA in the pediatric population is limited, but shows similarities with our findings. Two studies in infants aged 0–6 months observed that both methods generated highly correlated but significantly different absolute results [29,30]. In particular, FM and FM% estimates by ADP were significantly lower compared to DXA, while FFM results were higher; as also observed in present study. In adolescence, ADP and DXA results were reported to be strongly correlated [20,31]. However, FM% results were not comparable in subjects with more deviant body composition, such as individuals with severe under- or overweight [20,31]. These findings correspond with the observed proportional bias, as well as the larger inter-method differences in very preterm-born children. We extend the previous literature by adding data on ADP and DXA comparison in young children aged 3–5 years, in whom comparative studies were lacking.

Although DXA and ADP have been validated against four-component models in small samples of healthy children with normal weight [13], both machines use different techniques with limitations that could explain the observed differences. ADP has several limitations. It measures body volume using the inverse pressure-volume relation, which is sensitive for environmental factors that influence air pressure and density, such as crying of the subject or fluctuations in room temperature [10,13]. In addition, in order to calculate body composition parameters based on body volume, it uses density models that are based on multiple assumptions [10]. First, FM is thought to contain no water and have a constant density throughout life, whereas Dffm is considered to increase with age, as FFM hydration decreases throughout life [14,15,16]. Other assumptions include the content of bone mineral constituents and the amount of fat in the bones, as well as lung volume [10]. The Lohman Dffm model, used as default in the ADP software, was extrapolated from data of small populations of subjects aged 0–1 and 8–30 years measured in the 1980′s [14,15,16]. Dffm estimates were then extrapolated to other pediatric age categories per 2-year intervals [14]. Additionally, Dffm can vary in children with different nutritional status (e.g., hydration status), physical activity level, ethnicity, and disease status, but these variables were not included in the density models [14,32]. All these factors could have added to the inter-method differences observed in present study. DXA is based on a three-compartment model and uses the attenuation of X-ray energy passing different types of tissue [33,34]. DXA-software differentiates bone, fat, and other tissues. For pixels that contain mixed tissues, the software calculates the three parameters based on fixed algorithms using bone-edge detection [33]. These tools are based on a constant hydration status of FFM but it is known that the hydration status of FFM in children decreases with age [35]. Furthermore, it has been reported that DXA software encounters difficulties differentiating tissues in objects with a smaller body size [36]. DXA software might, therefore, be less accurate in young children, despite pediatric software options.

Our revised Dffm estimates are higher compared to those of Lohman et al. [14], which are used as default in the BODPOD machine for age 3–5 years. Our estimates are in line with a study from Wells et al. [16], who revised Dffm estimates for children aged ≥5 years using a four-compartment model. The Wells et al. estimates were more accurate compared to Lohman’s estimates in healthy 5.5-year-old children, when validated against a three-compartment model, including isotope dilution [17]. We have now added revised Dffm estimates for younger children, aged 3–5 years.

We observed that the inter-method differences were significantly larger in children born preterm compared to full-term children. Although using our revised Dffm estimates improved ADP results, considerable bias, fixed, and proportional, remained present. This could have several explanations. First, very preterm-born children are prone to experience impaired growth resulting in smaller body size as compared to full-term born peers [37], as also seen in our cohort. The aforementioned limitations of DXA software in subjects with small body size may, therefore, hamper accurate assessment of body composition in this group [36]. More importantly, children born very preterm show a different pattern of body composition and Dffm over childhood. While FM in preterm children was observed to be higher around term age, studies later in childhood reported lower FM and FFM as compared to full-term-born children [5,38]. Furthermore, recent studies showed that bone mineral content and density were also lower in preterm-born children at the age of 5–9 years as compared to full-term-born children [39,40]. Moreover, incorrect assumptions about thoracic gas volume could lead to incorrect body composition estimates by ADP [10]. Preterm-born children, with or without bronchopulmonary dysplasia (BPD), more often have reduced lung volumes or impaired lung function in mid childhood [41]. All these variables may complicate accurate assessment of body composition by ADP in the preterm population, in which accurate information on body composition is important for long-term health. Given the observed proportional bias between ADP and DXA, a low FM% will lead to greater inter-method differences. Moreover, a recent study in over 900 subjects aged 4–22 years showed that leaner body types have lower FFM hydration and consequently higher Dffm [32]. Altogether, we suggest that caution is needed when interpreting ADP results of this specific patient group. In fact, it warrants further research to compose separate Dffm estimates for children with deviant body composition trajectories, such as preterm-born children.

Strength of this study is the relatively large number of healthy full-term-born children who underwent an ADP and DXA assessment within one hour. To our knowledge, this is the first study to provide revised Dffm models for ADP in full-term-born children aged 3–5 years. Furthermore, comparing results with a group of very preterm-born children emphasizes the challenges of assessing body composition in children at risk for deviant growth patterns. We also acknowledge several limitations. We revised the Dffm model using FM% prediction not from a four-compartment but from a three-compartment model as reference method. However, a four-compartment model has not been investigated in children below the age of 5.5 years [34]. Although we observed improvement of ADP results using our revised Dffm estimates, future studies should explore how the revised estimates hold in pediatric populations elsewhere. In particular, including sufficient numbers of children from different ethnical groups would increase external generalizability. Moreover, our findings suggest the need for specific Dffm models for different patient groups with deviant body composition trajectories, such as preterm-born children. Because the three-compartment model was validated for healthy subjects, it proved not suitable as a reference for the very preterm group in our study. Further research, using a four-compartment model including isotope dilution, in larger cohorts is needed to calculate and validate Dffm estimates for particular patient groups (e.g., other growth disorders). Lastly, development of cheaper but reliable methods also applicable in lower-resource settings could improve body composition measurement in a broader sense.

## 5. Conclusions

Despite revised and improved age and sex-specific Dffm estimates for ADP, the results of ADP and DXA remained not comparable and should not be used interchangeably in the longitudinal assessment of body composition in children aged 3–5 years, especially in the case of very preterm-born children of that age.

## Figures and Tables

**Table 1:** Clinical characteristics.

	Full-Term		Very Preterm		*p*-Value
	Boys	Girls	Boys	Girls	
**Birth**	N = 79	N = 75	N = 39	N = 28	
Gestational age (weeks)	39.47 (1.29)	39.77 (1.24)	27.50 (1.55)	27.44 (1.55)	**<0.001**
Birth weight SDS	0.39 (1.00)	0.19 (1.09)	0.27 (0.68)	0.05 (0.76)	0.416
BPD (%)	*NA*	*NA*	12 (30.8%)	5 (17.9%)	
Ethnicity (%)					**<0.020**
White	54 (68.4%)	45 (60.0%)	30 (76.9%)	23 (82.1%)	
Non-white	25 (31.6%)	30 (40.0%)	9 (23.1%)	5 (17.9%)	
**All visits, total group**					
Weight-for-height SDS	0.07 (1.11)	0.40 (0.91)	−0.55 (1.10)	−0.51 (1.13)	**<0.001**
Height SDS	−0.26 (0.79)	−0.20 (1.02)	−0.87 (0.76)	−0.70 (1.11)	**<0.001**
** Age 3 years**	N = 18	N = 24	N = 13	N = 10	
Age (years)	3.06 (0.11)	3.08 (0.10)	3.44 (0.15)	3.46 (0.18)	**<0.001**
Weight-for-height SDS	0.31 (1.08)	0.51 (1.02)	−0.43 (0.94)	−0.47 (0.95)	**0.001**
Height SDS	−0.13 (0.72)	0.09 (0.93)	−0.69 (0.65)	−0.58 (1.34)	**0.008**
** Age 4 years**	N = 33	N = 24			
Age (years)	4.11 (0.13)	4.15 (0.15)	*NA*	*NA*	
Weight-for-height SDS	−0.13 (1.21)	0.45 (0.83)	*NA*	*NA*	
Height SDS	−0.32 (0.89)	0.02 (1.08)	*NA*	*NA*	
** Age 5 years**	N = 46	N = 41	N = 26	N = 18	
Age (years)	5.11 (0.14)	5.08 (0.13)	5.97 (0.17)	5.94 (0.12)	**<0.001**
Weight-for-height SDS	0.13 (1.05)	0.31 (0.14)	−0.61 (1.18)	−0.53 (1.25)	**<0.001**
Height SDS	−0.27 (0.75)	−0.51 (0.98)	−0.96 (0.80)	−0.77 (1.00)	**0.002**

Data are expressed as absolute numbers (percentage) or mean (SD). *p*-values represent the differences between full-term and very preterm-born children (both sexes combined), analyzed with independent *t*-test. Significant *p*-values are boldfaced. Abbreviations: n, number; SDS, standard deviation score, NA, not applicable; BPD, bronchopulmonary dysplasia.

**Table 2 jcm-11-01604-t002:** Body composition parameters assessed by ADP and DXA.

	Full-Term N = 186	Very Preterm N = 67	*p*-Value
FM (kg)			
DXA	5.16 (1.26)	4.43 (1.26)	**<0.001**
ADP default	4.09 (1.45)	2.54 (1.35)	**<0.001**
ADP revised	4.47 (1.40)	2.98 (1.73)	**<0.001**
Mean difference (LoA) ADP default–DXA	−1.08 * (−2.92; 0.76)	−1.89 * (−4.10; 0.32)	**<0.001**
Mean difference (LoA) ADP revised–DXA	−0.67 * (−2.38; 1.04)	−1.45 * (−3.53; 0.63)	**<0.001**
**FM%**			
DXA	28.26 (4.88)	24.39 (4.76)	**<0.001**
ADP default	22.47 (6.91)	14.60 (7.88)	**<0.001**
ADP revised	24.90 (6.64)	17.07 (7.93)	**<0.001**
Mean difference (LoA) ADP default–DXA	−5.78 * (−16.25; 4.69)	−9.79 * (−20.92; 1.34)	**<0.001**
Mean difference (LoA) ADP revised–DXA	−3.54 * (−13.44; 6.36)	−7.32 * (−18.26; 3.62)	**<0.001**
**FFM (kg)**			
DXA	13.06 (2.01)	13.72 (2.59)	0.064
ADP default	13.96 (2.25)	15.36 (3.36)	**0.002**
ADP revised	13.41 (2.13)	14.91 (3.28)	**0.001**
Mean difference (LoA) ADP default–DXA	0.90 * (−1.00; 2.80)	1.64 * (−0.63; 3.91)	**<0.001**
Mean difference (LoA) ADP revised–DXA	0.50 * (−1.30; 2.30)	1.20 * (−0.92; 3.32)	**<0.001**

Data are expressed as mean (SD). *p*-value term vs. preterm is difference between mean difference in term and very preterm-born children. * Indicates differences between ADP and DXA *p* < 0.001. Abbreviations: ADP, air-displacement plethysmography; DXA, dual-energy X-ray absorptiometry; N = number; FM = fat mass; FM% = fat mass percentage; FFM, fat-free mass; LoA, limits of agreement (95% CI).

**Table 3:** Revised fat-free mass density models for children aged 3–5 years.

Age (years)	Boys			Girls		
	C1	C2	Dffm	C1	C2	Dffm
2.75	5.432	5.031	1.0797	5.449	5.050	1.0790
3	5.424	5.022	1.0801	5.426	5.025	1.0800
3.25	5.416	5.013	1.0804	5.405	5.001	1.0808
3.5	5.409	5.005	1.0807	5.393	4.987	1.0813
3.75	5.402	4.998	1.0809	5.386	4.980	1.0816
4	5.395	4.990	1.0812	5.384	4.978	1.0816
4.25	5.390	4.984	1.0814	5.384	4.978	1.0816
4.5	5.386	4.980	1.0816	5.384	4.978	1.0816
4.75	5.384	4.978	1.0817	5.384	4.978	1.0816
5	5.384	4.977	1.0817	5.384	4.978	1.0816

Median Dffm and C1 and C2 predicted in 0.25-year intervals for children aged 3–5 years. C1 = (Dffm * Dfm)/(Dffm − Dfm). C2 = Dfm/(Dffm − Dfm)) Abbreviations: Dffm = fat-free mass density. Dfm= fat mass density = 0.9007 kg/L.

## Supplementary Materials

The following supporting information can be downloaded at: https://www.mdpi.com/article/10.3390/jcm11061604/s1, Table S1. Body composition parameters assessed by ADP and DXA between age 3–5 years.
