# Supplementary material for: Body Composition Assessment by Air-Displacement Plethysmography Compared to Dual-Energy X-ray Absorptiometry in Full-Term and Preterm Aged Three to Five Years
Source: J Clin Med. 2022 Mar 14;11(6):1604. doi: 10.3390/jcm11061604 (PMC8952802; doi:10.3390/jcm11061604)
Supplement: Supplementary file 1 [file jcm-11-01604-s001.zip › jcm-1570184-supplementary.pdf]

Supplemental Table S1. Body composition parameters assessed by ADP and DXA between age 3-5 years.

	**3 years**			**4 years**	**5 years**		
	Full-term	Very preterm	*p*-value	Full-term	Full-term	Very preterm	*p*-value
**FM (kg)**							
DXA	4.86 (1.20)	3.92 (0.79)	**0.001**	5.10 (1.21)	5.35 (1.30)	4.70 (1.39)	**0.009**
ADP default	3.67 (1.47)	3.34 (1.04)	0.344	4.16 (1.39)	4.24 (1.46)	2.12 (1.31)	**<0.001**
ADP revised	4.04 (1.46)	3.71 (1.03)	0.341	4.59 (1.42)	4.64 (1.32)	2.60 (1.39)	**<0.001**
**FM%**							
DXA	30.30 (4.76)	26.06 (4.01)	**0.001**	28.70 (4.86)	26.98 (4.60)	23.52 (4.93)	**<0.001**
ADP default	23.06 (7.65)	22.30 (5.69)	0.677	23.50 (6.66)	21.52 (6.65)	10.58 (5.52)	**<0.001**
ADP revised	25.53 (8.37)	25.05 (6.86)	0.815	26.01 (7.41)	22.87 (7.78)	10.96 (5.93)	**<0.001**
**FFM (kg)**							
DXA	11.07 (1.15)	11.11 (1.48)	0.893	12.56 (1.51)	14.35 (1.68)	15.08 (1.19)	**0.028**
ADP default	12.00 (1.32)	11.52 (1.36)	0.169	13.35 (1.67)	15.31 (2.05)	17.36 (2.10)	**<0.001**
ADP revised	11.63 (1.27)	11.15 (1.34)	0.157	12.92 (1.61)	14.73 (1.99)	16.88 (2.02)	**<0.001**

Data are expressed as mean (SD). P-value term vs preterm is difference between mean difference in term and very preterm born children. Abbreviations: ADP, air-displacement plethysmography; DXA, dual energy X-ray absorptiometry; FM, fat mass; FM% = fat mass percentage; FFM, fat-free mass; LoA, limits of agreement (95% confidence interval).
